# Efficacy and safety of limb lengthening in achondroplasia: A systematic review and meta-analysis

**DOI:** 10.1007/s00264-025-06720-z

**Published:** 2026-03-16

**Authors:** Gamal Hosny, Ahmed AbdElnaser, Abdelrahman Elashhab, Hossam Saad

**Affiliations:** Benha University, Benha, Egypt

**Keywords:** Achondroplasia, Limb lengthening, External fixator, Complications, Quality of life

## Abstract

**Purpose:**

To systematically review the efficacy, safety, and outcomes of limb lengthening procedures in patients with achondroplasia, including effects on quality of life.

**Methods:**

Following PRISMA guidelines, a systematic review and meta-analysis was performed. Eligible studies included patients with achondroplasia who underwent limb lengthening of the upper and/or lower extremities. Data were extracted on length gain, external fixator index, fixation duration, complications, and quality of life. Pooled means and 95% confidence intervals (CIs) were calculated using single-arm meta-analysis.

**Results:**

Fourteen studies including 1149 patients were analyzed. The mean femoral gain was 8.85 cm (95% CI: 7.42–10.28), tibial gain 7.36 cm (95% CI: 6.21–8.52), and humeral gain 8.38 cm (95% CI: 7.01–9.74). The mean fixator index was 37.1 days/cm (95% CI: 32.37–41.82), with a mean fixation duration of 7.71 months (95% CI: 5.98–9.63). The overall complication rate was 56.1% (95% CI: 26.9–85.2). Importantly, the pooled quality of life score measured by the Paediatric Quality of Life Inventory was 75.69 (95% CI: 65.14–86.23), indicating moderate improvement despite high treatment burden.

**Conclusion:**

Limb lengthening in achondroplasia achieves significant stature and proportional gains but requires prolonged treatment and carries a high complication risk. Nevertheless, improvements in functional ability and quality of life are evident, particularly when multi-limb lengthening is performed. Future studies should standardize outcome reporting, assess long-term QoL trajectories, and evaluate newer technologies such as intramedullary nails combined with multidisciplinary support.

## Introduction

Achondroplasia is the most common form of skeletal dysplasia, with an estimated incidence of approximately 1 in 20,000 to 30,000 live births worldwide. It is caused by activating mutations in the fibroblast growth factor receptor 3 (FGFR3) gene, which result in impaired endochondral ossification, particularly affecting the long bones [1]. Clinically, achondroplasia is characterized by rhizomelic limb shortening, macrocephaly, midface hypoplasia, and disproportionate short stature, with average adult heights of 131 ± 5 cm for males and 124 ± 5 cm for females [2]. In addition to the physical characteristics, patients often face orthopaedic complications such as genu varum, spinal stenosis, and recurrent otitis media, as well as psychosocial challenges related to reduced stature and body image [3].

Limb lengthening has been developed as a surgical strategy to increase height and improve limb proportions in patients with achondroplasia. The method is based on the principle of distraction osteogenesis, initially pioneered by Ilizarov in the mid-twentieth century, which involves gradual mechanical distraction of bone following a corticotomy, with new bone formation occurring in the distraction gap [4]. Over the last few decades, both external fixators (circular, monolateral, and hexapod devices) and, more recently, intramedullary lengthening nails have been used to achieve height gain in affected patients [5].

The reported benefits of limb lengthening extend beyond increased stature, including improvements in functional capacity, reach in daily activities, and self-perception [6]. However, the procedure is lengthy, technically demanding, and not without risks. Documented complications include pin site infections, joint contractures, nerve palsies, delayed union or nonunion, and psychological distress during the prolonged treatment course [7, 8]. In addition, there is an ongoing debate about the ethical implications of surgically altering stature in children, balancing medical necessity with psychosocial benefits [9].

While individual centres have published data on outcomes of limb lengthening in achondroplasia, the literature is fragmented, with considerable heterogeneity in patient selection, surgical techniques, and outcome reporting. Some studies have focused exclusively on lower limb lengthening (femur and tibia), while others have included upper limb procedures (humerus) to address the disproportionate shortening of the arms [10]. Furthermore, outcome measures vary widely, from objective parameters such as length gained and healing index to more subjective measures such as quality of life and psychosocial adjustment [11, 12].

There remains a critical need for a comprehensive synthesis that evaluates the efficacy and safety of limb lengthening in achondroplasia, with a focus on both functional and psychosocial outcomes. Such a review is essential to guide patient counseling, surgical planning, and multidisciplinary management. The present systematic review and meta-analysis aimed to address this gap by summarizing the available evidence on limb lengthening procedures in patients with achondroplasia.

## Materials and methods

This systematic review was conducted in accordance with the Preferred Reporting Items for Systematic Reviews and Meta-Analyses (PRISMA) guidelines [13].

### Eligibility criteria

- Inclusion criteria:
  o Population: Studies including patients with a confirmed diagnosis of achondroplasia.
  o Intervention: Limb lengthening procedures involving upper and/or lower limbs.
  o Outcomes: Reported increase in limb length, healing index, complications, and quality-of-life measures.
  o Study design: Prospective and retrospective cohort studies, case–control studies, and case series.
  o Language: English-language publications.
- Exclusion criteria:
  o Non-English studies.
  o Non-surgical interventions (e.g., growth hormone therapy).
  o Case reports.
  o Studies on other skeletal dysplasias without separate achondroplasia subgroup data.
  o Review articles, guidelines, book chapters, and abstracts without original data.

### Search strategy and study selection

Electronic databases (PubMed, Scopus, and Web of Science) were systematically searched up to July 2025 using a combination of keywords: achondroplasia, limb lengthening, distraction osteogenesis, Ilizarov, external fixator, and intramedullary nail. Titles, abstracts, and full texts were independently screened by two reviewers, with discrepancies resolved by consensus.

### Data extraction and synthesis

From each included study, data were extracted on study design, sample size, demographics, type of surgery, device used, outcomes including gain of length of tibia, femur, and humerus, external fixator index, duration of fixation, and complications.

### Quality assessment

For observational studies, the Newcastle–Ottawa Scale (NOS) was applied [14], assessing selection, comparability, and outcome domains. Studies scoring ≥ 7 on NOS were considered high quality.

### Statistical analysis

The process of statistical analysis was conducted as a single-arm analysis using Open Meta Analyst software. For continuous variables, the overall mean and 95% confidence interval (CI) was calculated and for dichotomous variables, the overall incidence and 95%CI was calculated.

## Results

### Searching and screening

**Fig. 1:**
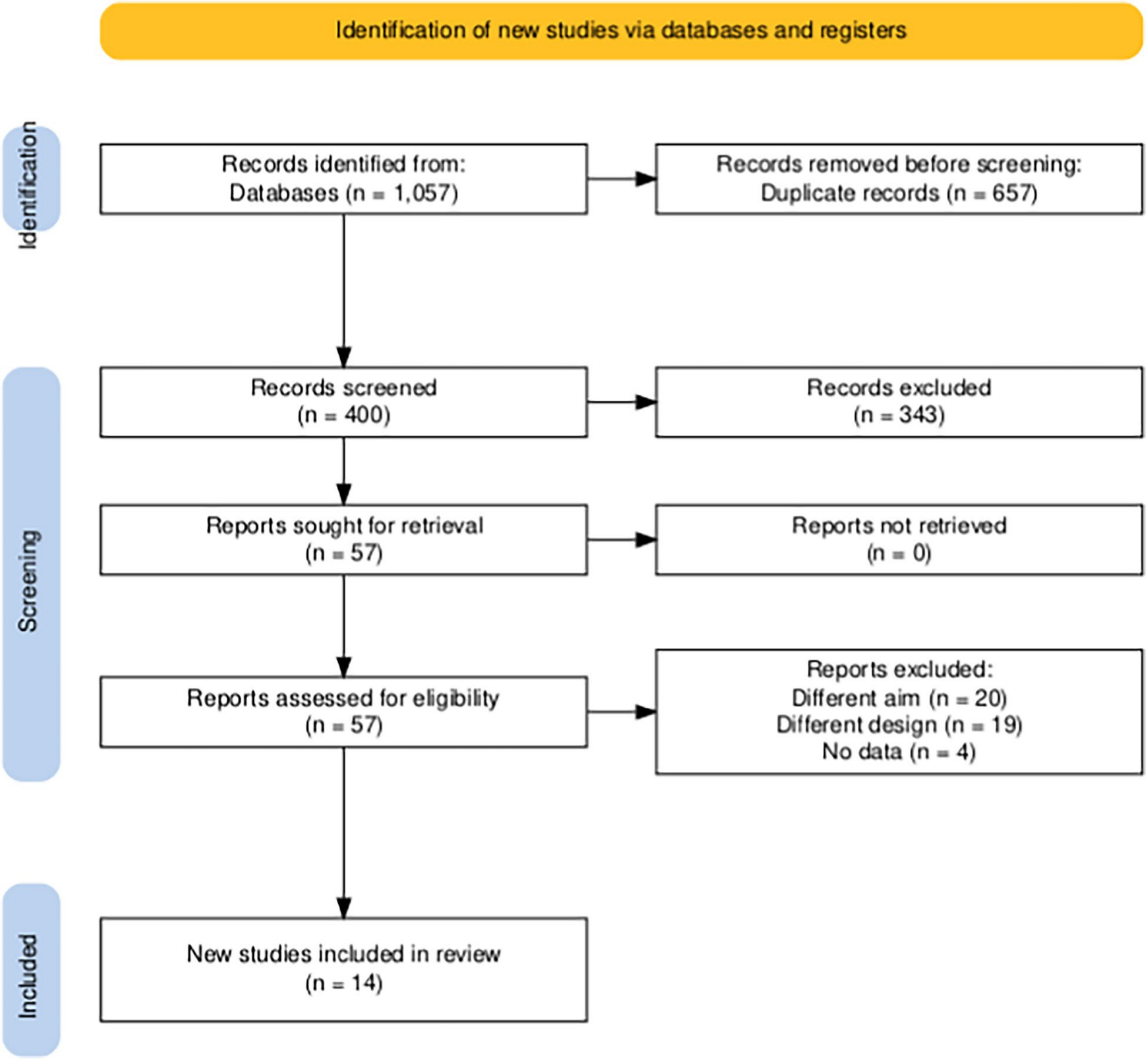
PRISMA flow diagram of searching and screening

The search yielded 1057 articles, of which 657 were identified as duplicates and removed. The remaining 400 articles underwent title and abstract screening. Following this, 57 full-text articles were assessed for eligibility, resulting in the inclusion of 14 studies in the final systematic review and meta-analysis [7, 8, 10–12, 15–23]. (Fig. 1).

### Baseline characteristics

A total of 14 studies met the inclusion criteria, comprising 1149 patients with achondroplasia who underwent limb lengthening procedures. The majority were retrospective or prospective cohort studies, with one case–control design. Study sizes varied considerably, from as few as eight patients [20] to as many as 750 patients in the large multicenter series [17]. The mean age at surgery ranged widely, from 6.17 years [11] to 20 years [20], reflecting the variability in timing of surgical intervention across centres. Most studies involved children and adolescents, although a subset included young adults. Gender distribution was relatively balanced overall, with male proportions ranging from 25 to 75%. Regarding surgical interventions, the techniques employed demonstrated significant heterogeneity. The Ilizarov circular fixator and its modifications were the most commonly utilized devices, particularly in earlier and larger studies [7, 17]. Monolateral external fixators were also frequently used, either alone or in combination with circular frames [16, 22]. More recent cohorts have reported the use of hexapod external fixators and hybrid systems that allow for multiplanar corrections and increased precision [15]. Both lower limb lengthening (femur and tibia) and upper limb procedures (particularly humeral lengthening) were represented in the literature. While femoral and tibial lengthening were most frequently performed, humeral lengthening was specifically addressed in other studies [10, 11], reflecting growing interest in improving upper limb functionality in addition to stature. Simultaneous bilateral procedures were reported in several studies, most notably by Shabtai et al. [12], who evaluated combined femoral and tibial lengthening, and by Leiva Gea et al. [23], who described bilateral simultaneous femoral and tibial surgeries. The breadth of surgical approaches highlights the diversity of practice patterns and evolving strategies to maximize both height gain and functional improvements. (Table 1).

**Table 1:** Baseline characteristics of the included studies

Study ID	Design	Sample size	Age, mean (SD)	Male, n (%)	Operation
Bocchi 2025	Cohort	14	11.3 (2.15)	7 (50)	Circular external fixator or Hexapod external fixator
Venkatesh 2009	Cohort	20	12.35 (3.8)	5 (25)	Bilateral femoral lengthening using a monolateral external fixator
Balci 2015	Cohort	18	10 (2.5)	8 (44.4)	Bilateral humeral lengthening using monorail external fixators
Park 2015	Cohort	28	14.3 (4.2)	10 (35.7)	Bilateral limb lengthening using Ilizarov ring fixator and monolateral external fixator
Diachkova 2018	Cohort	750	4 to 23	353 (47)	Bilateral limb lengthening using Ilizarov method
Song 2012	Cohort	35	8.2 (1.96)	17 (48.6)	Bilateral limb lengthening
Batibay 2020	Case–control	49	6.17 (2.7)	20 (40.8)	Bilateral limb lengthening with or without humeral lengthing
Bayram 2022	Cohort	95	8.78 (3.74)	35 (36.8)	Limb lengthening surgery for both upper and lower extremities
Shabtai 2021	Cohort	50	11.8 (4.9)	29 (58)	Simultaneous bilateral femoral and tibial lengthening procedures
Verdoni 2023	Cohort	33	8.73	12 (36.4)	Bilateral limb lengthening
Lie and Chow 2009	cohort	8	20 (10.5)	6 (75)	Limb lengthening using monolateral and circular external fixators
Trofimchuk 2024	Cohort	14	7.6 (2.3)	8 (57.14)	Paired limb lengthening with an external fixator
Devmurari 2010	Cohort	14	10 (1.8)	6 (42.8)	Femoral lengthening using a monolateral fixator
Leiva-Gea 2020	Cohort	21	11.76 (1.89)	12 (57.14)	Bilateral simultaneous surgery of the femur and tibia

SD: standard deviation

### Quality assessment

All the included studies were deemed to be of moderate quality (4–6 stars) according to NOS measurement. (Table 2).

**Table 2:** Quality assessment of the included studies using NOS

Study ID	Selection (max 4)	Comparability (max 2)	Outcome (max 3)	Total (max 9)
Bocchi 2025	☆☆☆	-	☆☆☆	☆☆☆☆☆☆
Venkatesh 2009	☆☆☆	-	☆☆☆	☆☆☆☆☆☆
Balci 2015	☆☆	-	☆☆☆	☆☆☆☆☆
Park 2015	☆☆	-	☆☆☆	☆☆☆☆☆
Diachkova 2018	☆☆	-	☆☆☆	☆☆☆☆☆
Song 2012	☆☆☆	-	☆☆☆	☆☆☆☆☆☆
Batibay 2020	☆☆☆	-	☆☆☆	☆☆☆☆☆☆
Bayram 2022	☆☆	-	☆☆☆	☆☆☆☆☆
Shabtai 2021	☆☆	-	☆☆	☆☆☆☆
Verdoni 2023	☆☆	-	☆☆☆	☆☆☆☆☆
Lie and Chow 2009	☆☆☆	-	☆☆☆	☆☆☆☆☆☆
Trofimchuk 2024	☆☆☆	-	☆☆	☆☆☆☆☆
Devmurari 2010	☆☆☆	-	☆☆	☆☆☆☆☆
Leiva-Gea 2020	☆☆☆	-	☆☆	☆☆☆☆☆

### Statistical analysis

**Fig. 2:**
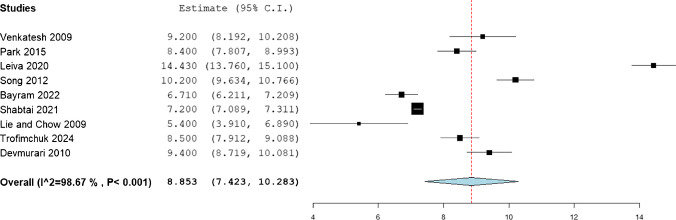
Mean femoral gain in the included patients

**Fig. 3:**
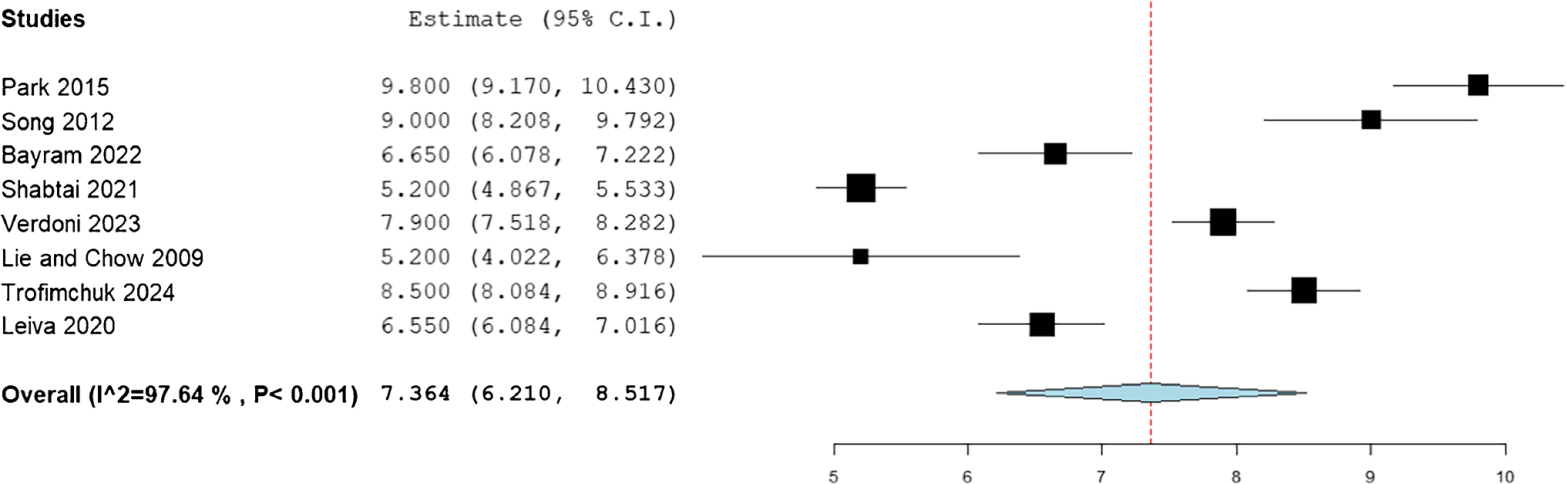
Mean tibial gain in the included patients

**Fig. 4:**
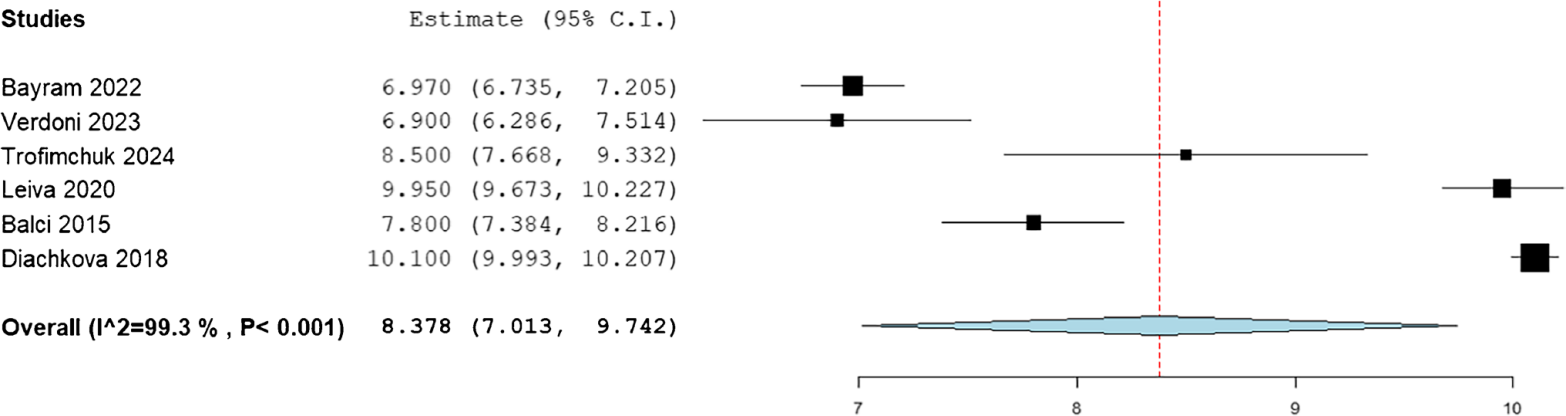
Mean humeral gain in the included patients

The statistical analysis of mean gain in the femoral length was observed to be 8.853 (95%CI: 7.423, 10.283), while that of tibia was 7.364 (95%CI: 6.21, 8.517), and that of humerus was 8.378 (95%CI: 7.013, 9.742). (Figs. 2, 3, 4).

**Fig. 5:**
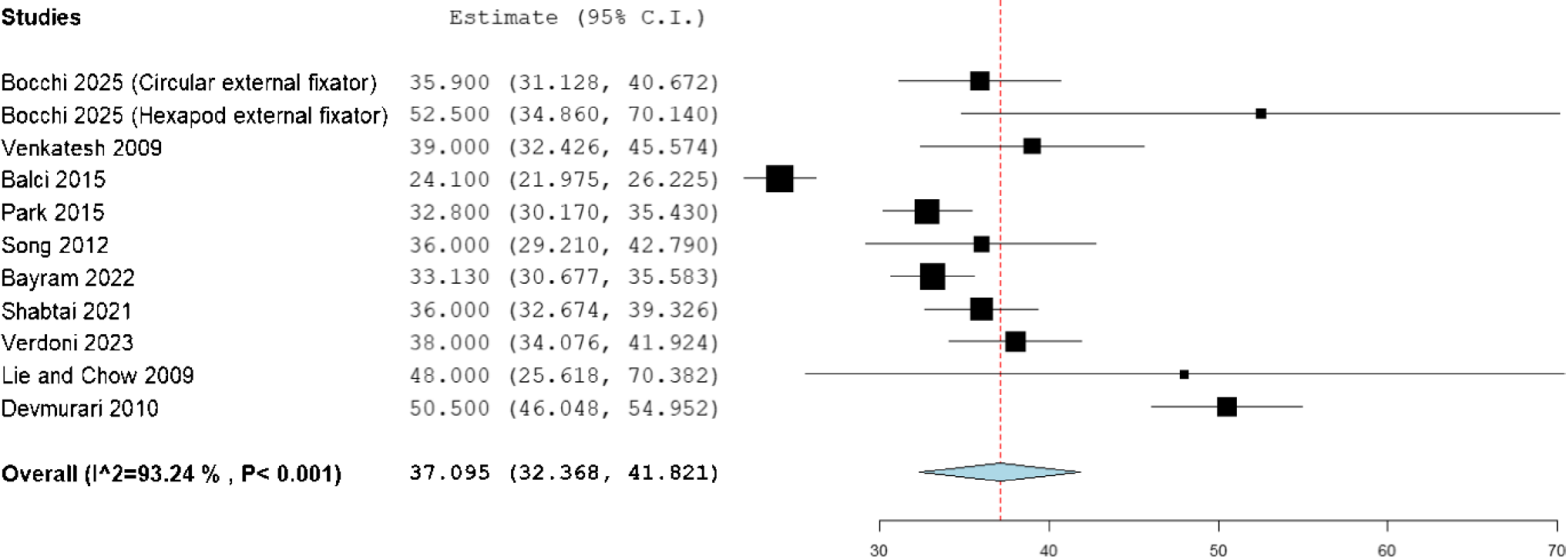
Mean fixator index in the included patients

**Fig. 6:**
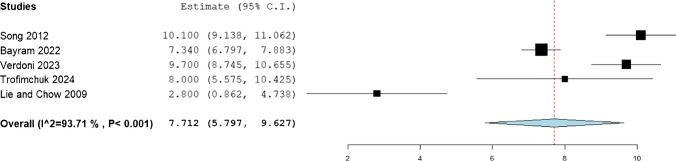
Mean duration of fixation in the included patients

The mean fixator index was observed to be 37.095 (95%CI: 32.368, 41.821) days, while the mean duration of fixation was 7.712 (95%CI: 5.979, 9.627) months. (Figs. 5 and 6).

**Fig. 7:**
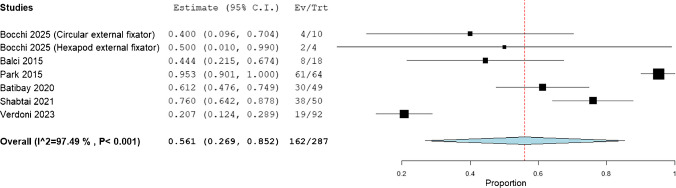
Incidence of complications in the include patients

**Fig. 8:**
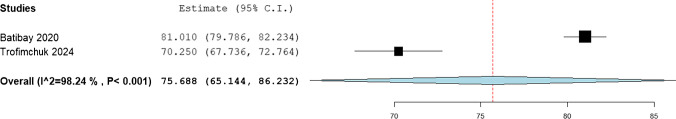
Overall quality of life measured by Paediatric Quality of Life Inventory

The incidence of complications was observed to be 56.1% (95%CI: 26.9%, 85.2%) and the overall quality of life measured by Paediatric Quality of Life Inventory was observed to be 75.688 (95%CI: 65.144, 86.232). (Figs. 7 and 8).

## Discussion

### Summary of findings

The present systematic review synthesized data from published series of limb lengthening in achondroplasia and found that mean gains in bone length were substantial but came with notable treatment burdens. Across included studies, patients achieved on average 8.85 cm of femoral lengthening, 7.36 cm for the tibia, and 8.38 cm for the humerus, using primarily external fixator techniques. The mean duration in fixation was long (7.71 months) and the fixator index – time in frame per centimeter lengthened – was 37.1 days/cm. The overall rate of complications (including “problems”, “obstacles” and true complications by Paley’s classification) was high (56.1% of patients or segments, as reported). These findings indicate that, in achondroplastic patients, appreciable height and proportion gains are achievable but require prolonged treatment and carry a high incidence of difficulties.

### Investigation with previous literature

For context, other series report broadly similar limb gains: for example, Park et al. [7] reported mean gains of 8.4 cm (femur) and 9.8 cm (tibia) using staged bilateral lengthening, very close to our pooled femur and tibia means. Other authors report even larger gains in some bones as Chilbule et al. [5] found up to 15.4 cm tibial lengthening (with bifocal corticotomies), but also note a higher rate of complications with such aggressive strategies. In humeral lengthening, our mean (8.38 cm) was likewise in the range reported by isolated series such as that by Chilbule et al. [5] (9.6 cm), though few large cohorts focus solely on upper limbs.

Our observed fixator index of 37.1 days/cm is somewhat higher than the healing indices reported in intensive protocols. For example, Chilbule et al. [5] reported tibial/femoral healing indices of ~ 25 days/cm, likely reflecting simultaneous bifocal lengthening. By comparison, Park et al. [7] observed tibial and femoral external fixator index to be comparable to values reported in the literature for lengthening in healthy bone [24, 25]. The mean fixation time of ~ 7.7 months in our data is similar to times reported elsewhere (e.g. ~ 9–10 months for femurs in one large series) [19], underscoring the extended treatment courses involved.

Notably, the pooled complication rate (56.1%) is very high but aligns with prior literature. In earlier reviews, complication frequencies (counting obstacles and problems) ranged from 46 to 72% [26–28], so our result is within this range. Some series report much lower major complication rates: for example, Batıbay et al. [11] described only 20.7% complications over 92 lower-limb segments, but this likely reflects narrower reporting (excluding minor pin-site issues and temporary obstacles). In contrast, studies counting all Paley-style categories invariably find very frequent issues. Moreover, the literature consistently shows a higher complication burden in femoral lengthening than tibial: one group found refractures and angular deformities far more common in femoral osteotomies [7]. It was noted that tibial lengthening often has a faster regenerate and fewer refractures, which has led some experts to recommend performing tibial lengthening (with knee protection post-frame removal) before femoral lengthening [7].

When our results are placed in context with other skeletal dysplasias, the patterns are broadly similar, though data on non-achondroplasia dysplasias are sparse. Paley’s [29] review of achondroplasia/hypochondroplasia lengthening (which included nearly 200 patients) found average total gains of ~ 14–20 cm per patient, comparable to our findings, and highlighted that multi-stage four-segment protocols can achieve much larger total height gains (up to 30–40 cm) than the traditional two-segment approach. In summary, our efficacy results include moderate length gains but well below “normal” stature are consistent with prior reports: typical femur/tibia gains are on the order of 5–10 cm, and even extensive strategies rarely reach the hundreds of centimeters; this underlines why many “extensive” series aim for 30 + cm total height gain to approach normal adult height [29].

### Clinical implications

The findings of this review carry several implications for practice. This includes surgical planning and sequencing. Given the relative resilience of the tibia and the higher risk in the femur, most experts recommend staged lengthening typically bilateral tibiae first, then bilateral femora, and consider upper limbs as separate procedures if needed [7]. Treatment is typically spread over multiple operations to limit length per segment (often ~ 5–8 cm per operation) and to allow recovery. Younger patients (age ~ 6–8) are often selected for initial lengthening during the juvenile growth phase [19], allowing a protracted course of multi-stage lengthening during childhood. Lower limb alignment issues (e.g. genu varum or valgum) can often be corrected concurrently with the lengthening frames, offering a dual benefit. This also includes patient selection as achondroplasia patients considered for lengthening should be carefully evaluated. Ideal candidates are those with significant psychosocial distress or functional impairment from short stature who understand the risks. Contraindications include significant spinal or cardiopulmonary comorbidities. The multidisciplinary setting is crucial as treatment decisions should involve genetics, paediatrics, orthopaedics, and psychology [19]. Comprehensive preoperative counseling is essential, emphasizing the expected amount of height gain (often well below average) and the high likelihood of secondary procedures (for complications or frame adjustments). For example, achieving just the low-normal height for sex requires on the order of 30–40 cm of total gain, far more than a single lengthening, so families must appreciate the multistage nature of “extensive” protocols [29].

The primary aim of lengthening in achondroplasia is not merely cosmetic; it is to improve reach, mobility, and life participation. Some evidence indicates real functional benefits – patients report easier dressing, standing, and social participation after lengthening [30]. Importantly, psychological outcomes appear linked to the degree of overall height gain: achieving a height above certain thresholds (e.g. ≥ 140 cm) correlates with better quality of life scores [26]. Nonetheless, the prolonged treatment can strain both patients and families. We reiterate reports that coordinated psychological support (including counseling and peer support) throughout the lengthening process is recommended for all patients [19]. Physical therapy is also key, to maintain joint motion and muscle strength during distraction, and orthotic or casting protocols should be used to prevent contractures and rebound deformities.

### Strengths and limitations

This systematic review is one of the first to aggregate contemporary outcomes of limb lengthening in achondroplasia. Strengths include a broad search of the literature and inclusion of multiple large series. However, several limitations must be acknowledged. First, the available studies are heterogeneous and mostly retrospective, with variable methodologies and outcome reporting. There were relatively few high-quality trials or registries; many studies have small sample sizes and institutional biases. Second, all included series focused on external fixation techniques; newer methods (e.g. motorized intramedullary nails or lengthening-over-nail) were not represented or were in preliminary stages. This external fixator-centric focus limits applicability as intramedullary nails become more common. Third, outcome measures varied – some authors report fixator index, others healing index, and few report standardized functional scores or patient-reported outcomes. There was no consistency in defining or grading complications across studies. These inconsistencies prevented pooling of certain outcomes (for example, consolidation times) and may have introduced bias in the complication rates. Finally, a major limitation is the lack of long-term follow-up data in most studies. As others have warned, the long-term effects of extensive lengthening (on joints, back, and overall musculoskeletal health) remain uncertain.

### Recommendations and future directions

Our review highlights several areas for improvement in both research and clinical practice. First, there is a need for standardized outcome reporting in achondroplasia lengthening. Authors should ideally adopt common measures (e.g. external fixator index, percent height gain, Paley’s classification of complications, and validated functional/quality of life instruments) to allow comparisons. Prospective registries or collaborative studies across centers would help generate higher-quality evidence. Second, as new technologies emerge, comparative studies are warranted. Motorized intramedullary nails offer the promise of eliminating external frames, potentially shortening treatment time and reducing superficial complications. Early reports suggest nails may yield fewer infections and less psychological stress, but their use in young achondroplasts is limited by bone size. Feasibility and outcomes of internal lengthening versus external fixator in achondroplasia should be investigated. Third, given the significant psychosocial burden, we recommend integrated psychosocial support as a standard part of care. Formal counseling and peer-group programs could be evaluated for benefit. Fourth, with new pharmacologic growth therapies (e.g. vosoritide) now available for achondroplasia, future research should address how these agents interact with surgical lengthening. It is conceivable that medical therapy could reduce the extent of surgery needed or improve regenerate quality. Finally, multidisciplinary care models involving orthopedists, endocrinologists, geneticists, physiotherapists and psychologists should be emphasized, as underscored by recent expert reviews. In practice, each decision for lengthening must balance the measurable gains in height and proportions against the substantial risks and burdens documented here.

## Conclusion

Limb lengthening in achondroplasia can achieve meaningful increases in stature and limb proportions, but it is a prolonged process with a high rate of complications. Our review found average length gains of ~ 8–9 cm in the femur, 7–8 cm in the tibia, and 8 cm in the humerus, requiring ~ seven to eight months in an external fixator per segment and resulting in complications in over half of patients. These outcomes are broadly consistent with prior series. Clinically, this underscores the importance of meticulous surgical planning (favoring staged tibial then femoral lengthening) and thorough patient/family counseling about realistic goals and risks. Functional and psychosocial benefits have been reported particularly when multiple limbs are lengthened, but the evidence is mixed. Our findings suggest that while lengthening can improve daily function and quality of life, these procedures should be undertaken within a multidisciplinary program with proactive rehabilitation and psychological support. For future practice, we advocate standardized outcome reporting and exploration of newer techniques (e.g. internal lengthening nails and combination with pharmacotherapy) to reduce treatment burden. Ultimately, the decision to perform limb lengthening should be individualized, weighing the potential stature and functional benefits against the long treatment course and high complication risk documented across the literature.

## Data Availability

No datasets were generated or analysed during the current study.
